# Supplementary material for: Impact of CD40 (rs1883832) and CD40L (rs1126535) gene variants on laryngeal cancer susceptibility and their association with serum biomarker levels of sCD40 and sCD40L
Source: PLoS One. 2024 Dec 3;19(12):e0312576. doi: 10.1371/journal.pone.0312576 (PMC11614260; doi:10.1371/journal.pone.0312576)
Supplement: Attachment — Submitted filename: Review comments.docx [file pone.0312576.s002.docx]

**Major Concerns:**

1. **Lack of Clarity in Results Interpretation:**

The paper presents the results in a somewhat fragmented manner. The relationship between the genotypes, sCD40, and sCD40L levels and their implications for laryngeal cancer susceptibility are not fully integrated. The discussion section should tie together the findings more cohesively to explain their relevance to the study’s hypothesis.

2. **Inadequate Explanation of Mechanisms:**

The paper hints at possible mechanisms by which CD40 and CD40L may influence cancer susceptibility, but these are not elaborated upon sufficiently. For example, the discussion on how the CD40 (rs1883832) polymorphism might alter protein function or immune response needs more depth, especially considering the observed lack of difference in sCD40 levels.

3. **Study Design Limitation:**

The study is limited by its focus solely on male participants, which is mentioned but not adequately addressed in the discussion of limitations. The lack of female participants significantly limits the generalizability of the findings. This should be acknowledged more explicitly, and the potential implications of gender differences in genetic susceptibility should be discussed.

4. **Unclear Rationale for Statistical Analysis Choices:**

The paper uses several statistical tests but does not always justify why specific tests were chosen or how they are appropriate for the data distribution. For instance, while the paper mentions that data followed a normal distribution, it does not explain why parametric tests were preferred or how outliers were handled beyond visual inspection.

**Minor Concerns:**

1. **Typographical and Grammatical Errors:**
   - There are several minor typographical and grammatical errors throughout the paper, such as "discrepancy" instead of "difference" and inconsistent use of terms like "CD40-producing cells" and "CD40L receptors." These should be corrected for clarity and consistency.
2. **Table and Figure References:**

The references to tables and figures in the text could be more precise. For example, "Table 1" and "Figure 1a" should be referenced at specific points where their content is discussed to aid the reader in following the results.

3. **Ambiguity in Some Sections:**

Some sections, particularly the "Materials and Methods" and "Results," are overly dense with technical jargon and could benefit from simplification or additional explanation to make them more accessible to a broader audience.

4. **Literature Review Depth:**
   - While the paper mentions that a thorough literature review was conducted, it might benefit from referencing more recent or diverse studies, particularly in the "Introduction" and "Discussion" sections. This would help frame the findings within the broader context of current research.
5. **Conclusion Section Needs Strengthening:**

The conclusion should more clearly summarize the key findings and their implications. The current conclusion is somewhat tentative and could be stronger in stating the study's contribution to understanding laryngeal cancer susceptibility.

6. **Formatting Consistency:**

Ensure that formatting is consistent, particularly in the presentation of gene names, statistical values, and references. For example, the notation of "sCD40" and "sCD40L" should be uniform throughout the paper.

Manuscript need thorough revision.
