# Supplementary material for: Impact of CD40 (rs1883832) and CD40L (rs1126535) gene variants on laryngeal cancer susceptibility and their association with serum biomarker levels of sCD40 and sCD40L
Source: PLoS One. 2024 Dec 3;19(12):e0312576. doi: 10.1371/journal.pone.0312576 (PMC11614260; doi:10.1371/journal.pone.0312576)
Supplement: Attachment — Submitted filename: responses to reviewer.docx [file pone.0312576.s003.docx]

**Responses to Reviewers**
**Manuscript ID:** PONE-D-24-12557
**Title:** Investigation of CD40, CD40L Gene Variants and sCD40, sCD40L Serum Levels in Laryngeal Cancer
**Date:** Sep 17, 2024
**To:** Academic Editor

Dear Reviewers,

We are grateful for the constructive comments and suggestions provided by the reviewers. We have carefully revised our manuscript to address all the points raised. Below is a point-by-point response to the reviewers' comments, along with details of the revisions made in the manuscript.

**Reviewer #1:**

**Comment 1: The manuscript requires major revisions, particularly in the Abstract and Conclusion, which should be revised in light of updated results and methodology sections.**

**Response:** We have revised the Abstract to provide clearer and more specific details regarding the statistical significance of the findings related to CD40 and CD40L gene variants and serum levels. The conclusion has also been updated.

**Comment 2: The introduction needs a more detailed explanation of the biological significance of CD40 and CD40L in cancer immunology.**

**Response:** We have expanded the introduction to include a more thorough explanation of the role of CD40 and CD40L in cancer immunology.

**Comment 3: More recent studies on CD40 and CD40L in various cancers should be included.**

**Response:** We have updated the literature review to include recent studies on CD40 and CD40L, particularly focusing on head and neck cancers. These additions provide a more comprehensive background and enhance the positioning of our study within the current research landscape.

**Comment 4: A clearer statement of the research gap addressed by the study is needed.**

**Response:** We have revised the introduction to emphasize the specific gap in the literature that our study addresses. The novelty of investigating CD40 and CD40L polymorphisms in laryngeal cancer is now highlighted more prominently.

**Comment 5: More details are required on patient exclusion criteria and sample size justification.**

**Response:** We have added further details on the criteria used for excluding patients in the Materials and Methods section. Additionally, we have included a rationale for the sample size, explaining that the number of patients and controls was determined based on power analysis, ensuring sufficient statistical power to detect significant differences.

**Reviewer #2:**

**Comment 1: The title could be more specific, and the abstract should clarify the statistical significance of findings.**

**Response:** We have revised the title as follows: *"* *Impact of CD40 (rs1883832) and CD40L (rs1126535) Gene Variants on Laryngeal Cancer Susceptibility and Their Association with Serum Biomarker Levels of sCD40 and sCD40L."*
Additionally, the abstract has been updated to better reflect the statistical significance of the findings and the most impactful results of the study.

**Comment 2: The statistical analysis needs more clarity, particularly regarding outliers and multiple comparisons.**

**Response:** We have provided additional information regarding how outliers were identified and handled in the statistical analysis. Moreover, we have clarified that corrections for multiple comparisons were applied, ensuring the robustness of our findings.

**Comment 3: The results and figures should avoid redundancy, and p-values should be reported consistently.**

**Response:** We have streamlined the presentation of results to avoid redundancy, and all p-values have been adjusted to be reported consistently to three decimal places. We have also specified whether the p-values are one-sided or two-sided.

**Additional Revisions in Response to Reviewers' Comments:**

1. **Grant Information:** We have corrected the discrepancies in the grant information between the “Funding Information” and “Financial Disclosure” sections. Additionally, we have provided the following statement regarding the role of the funders:
   *"The funders had no role in study design, data collection and analysis, decision to publish, or preparation of the manuscript."*
2. **Data Availability:** The Data Availability Statement has been revised to state:
   *"All data are in the manuscript and/or supporting information files."*
   This ensures compliance with PLOS ONE’s data-sharing policies.
3. **Ethics Statement:** The ethics statement has been removed from all sections except the Methods, in accordance with the reviewers' request.
4. **Supporting Information:** Captions for the Supporting Information files have been included at the end of the manuscript and corresponding in-text citations have been updated accordingly.
5. **Figures and Tables:** All figures and tables have been reviewed and clarified for better readability. We have uploaded the figures to the PACE tool as instructed and ensured they meet PLOS ONE’s technical requirements.

We hope that these revisions meet the expectations of the reviewers and the journal. We look forward to your feedback and thank you again for the opportunity to improve our manuscript.

Sincerely,
Dr. Alper Gümüş
Istanbul University
